# 

**Published:** 1916-11-11

**Authors:** 


### GIFTS AND BEQUESTS.

Mr. and Mrs. Player, of Clydach, Swansea Valley, have given £2,000 to Swansea General and Eye Hospital.

The Salisbury Infirmary has received £500 from the late Mr. T. K. Harding, of Ashton Gifford House, Codford St. Peter, Wilts.

A Cardiff coal exporter and a Cardiff shipowner have each given £5,000 as anonymous gifts to the proposed Welsh Hospital for Crippled Soldiers.

Mr. Lewis Lougher, of Cardiff, has sent a cheque for 1,000 guineas to the treasurer of King Edward VII.'s Hospital for the endowment of a bed in his name. Messrs. Furness, Withy and Co. have given, through Mr. W. H. Buckingham, 250 guineas to the hospital.

Miss Sarah Jane Tinker, of Thongsbridge, Huddersfield, left £500 each to Huddersfield Infirmary, the Royal Albert Institution for the Feeble Minded (Lancaster), and the Yorkshire School for the Blind.

The late Mr. Alfred Edmonds, of Wolverhampton, has left the following legacies to local charities: The General Hospital, £1,000; the Royal Orphanage, £1,000; the Counties Eye Infirmary, £500; the Hospital for Women, £500; the Society for the Blind, £500; the residue of his property to the Children's Hospital, the General Hospital, and the Queen's Hospital, Birmingham.

The board of management of the Manchester Royal Infirmary has received £1,000 from Mr. and Mrs. William Hughes, of Sale, to endow a bed in perpetuity in memory of their son, Rhys Hughes, who died of wounds in France on August 1, 1916. £1,000 has also been presented to the infirmary for the permanent endowment of a bed "In memory of Captain Edgar Kessler," who was killed in Gallipoli.

Under the will of the late Mr. Alexander Balfour, of Inchock, Arbroath Infirmary has been left £1,000 towards the building fund.

The Queen has sent a gift of grapes to the patients in the Royal Hospital for Incurables, Putney Heath, and also a parcel of silks and brocades for their use in making articles for the Annual Sale of Work.

The following Leeds hospitals receive £250 each under the will of the late Mr. J. C. Nicholson: General Infirmary, Women's and Children's Hospital.

The Queen has presented the Royal Pavilion Hospital at Brighton with a building to be used as a school for the teaching of some occupation to limbless soldiers.

Mr. C. J. Whitefield, of Cardiff, who died at Clifton on July 24, left £200 each to St. Mary's Convalescent Home, Clevedon, and St. Agnes' Home, Knowle, Bristol.

Mr. Hahnemann Epps, for many years managing director and chairman of Messrs. James Epps and Co., has left £5,000 to the Homœopathic Hospital, subject to the life interest of his wife and daughter.

Sir Michael Barker Nairn, Bt., has bequeathed £6,000 for the endowment fund of Kirkcaldy Hospital; £2,000 to the Cupar Town Council, the income to be equally divided between the Cupar Victoria Nursing Association, the Ladybank Victoria Nursing Association, the Letham Nursing Association, and for assisting nursing in the villages of Springfield and Pitlessie; £1,000 for the endowment fund of Dysart Victoria Nurses; £2,500 for the endowment fund of Kirkcaldy Victoria Nurses; and £500 to the Cupar Cottage Hospital for the endowment fund.

### Matrons' and Sisters' Appointments.

BOOKER, WYCOMBE ISOLATION HOSPITAL.—Miss Robinson has been appointed matron. She was trained at Southampton Borough Hospital, and has since been charge nurse at Guildford Joint Hospital and staff nurse at Dorking Joint Hospital and at Newbury Isolation Hospital.

HEYWOOD AUXILIARY HOSPITAL.—Miss S. Rhodes has been appointed matron. She was trained at St. Luke's Hospital, Bradford, and has since been ward sister and assistant matron at the Liverpool Convalescent Hospital, Woolton, Liverpool, and sister-in-charge at the Haye Leigh Hospital, Derby.

NORWICH ISOLATION HOSPITAL.—Miss Flora Morrison has been appointed matron. She was trained at the Royal Alexandra Hospital, Paisley, and has since been matron of the City Fever Hospital, Birmingham. She has been engaged in military nursing.

SOUTHPORT, THE CHILDREN'S HOME, MARSHSIDE ROAD.—Miss Charlotte Wakefield has been appointed matron. She was trained at Wolverhampton and Staffordshire General Hospital, and has since been sister at the Royal Salop Infirmary, Shrewsbury, sister-in-charge of the out-patient department at the Liverpool Infirmary for Children, night superintendent and assistant matron of the same institution, and matron of the Woodlands Convalescent Home and Auxiliary Military Hospital, Rawdon, Leeds.

WESTCLIFF-ON-SEA BOROUGH SANATORIUM FOR INFECTIOUS DISEASES.—Miss Florence Midgley has been appointed matron. She was trained at Halifax Royal Infirmary, and has since been deputy matron at the City Hospital Annexe, Fazakerley, Liverpool, and home sister at the Park Hospital, Hither Green.

HACKNEY UNION INFIRMARY.—Miss Annie Waterhouse and Miss Dora Dillon have been appointed sisters (charge nurses). Miss Waterhouse was trained at St. Luke's Hospital, Bradford, where she was afterwards charge nurse. She has also done private nursing, and holds the certificate of the Central Midwives Board. Miss Dillon was trained at Hackney Union Infirmary, where she has since been staff nurse.

HUDDERSFIELD WAR HOSPITAL.—Miss Strelley-Upton has been appointed night superintendent. She was trained at Great Yarmouth General Hospital, where she later became night sister, and has since been acting holiday sister at the Royal United Hospital, Bath; charge nurse at Gloucester Fever Hospital; sister at Hertford British Hospital, Paris; night sister at the Jenny Lind Hospital for Children, Norwich; and senior sister at Philadelphia General Hospital, U.S.A.

STAFFORD, SANDON HALL AUXILIARY MILITARY HOSPITAL.—Miss E. Smythe has been appointed sister. She was trained at Poplar Hospital, and has since been charge nurse at the Mount Vernon Hospital and sister-in-charge of Oswestry Auxiliary Military Hospital. She has also done private nursing.

ECCLESALL BIERLOW UNION INFIRMARY.—Miss Fanny T. Wallis has been appointed night superintendent. She was trained at Ecclesall Bierlow Union Infirmary, and has since been ward sister.

CITY OF WESTMINSTER UNION INFIRMARY, HENDON.—Miss Jessie McKelvin and Miss L. A. M. Smith have been appointed sisters. They were trained at St. Mary's Infirmary, Islington, and have since been staff nurses at the Norfolk War Hospital and at Croesnewydd Auxiliary Military Hospital, Wrexham.

AYLESBURY, ROYAL BUCKINGHAMSHIRE HOSPITAL.—Miss M. Waters has been appointed sister. She was trained at Princess Alice Hospital, Eastbourne, where she was later staff nurse

LIVERPOOL, NEW HOSPITAL, GARSTON.—Miss F. Maddock has been appointed sister. She was trained at the North Derbyshire Hospital, Chesterfield, and has since been staff nurse at Royal Infirmary, Blackburn, and theatre sister and ward sister at Woodlands V.A.D. Hospital, Southport. She has also done private nursing.

HIGHFIELD MILITARY HOSPITAL, LIVERPOOL.—Miss Edith E. Domville has been appointed theatre sister. She was trained at the Withington Hospitals, Manchester, and has since had varied nursing experience. Miss Domville holds the certificate of the Central Midwives Board.

**NOTE.—Particulars o Appointments for insertion in this column will be welcomed.**

RATES OF SUBSCRIPTION: Twelve months. 6/6; Six months, 4/-; Three months, 2/-; post free to any address in the United Kingdom. Abroad, Twelve months, 8/8: Six months, 5/-; Three months, 2/6, post free. THE HOSPITAL "Index" to Volume LX., April to September 1916, is now ready, price 2½d. per copy, post free. Address The Manager, "THE HOSPITAL," The Hospital Building, 28/29 Southampton Street, Strand, London, W.C.

